# Effect of maternal diet on gut bacteria and autism spectrum disorder in offspring

**DOI:** 10.3389/fncel.2025.1623576

**Published:** 2025-08-06

**Authors:** Zilin Chen, Xu Wang, Yuchen Hu, Si Zhang, Fei Han

**Affiliations:** ^1^Department of Pediatrics, Guang’anmen Hospital, China Academy of Chinese Medical Sciences, Beijing, China; ^2^State Key Laboratory of Traditional Chinese Medicine Syndrome, The Second Affiliated Hospital of Guangzhou University of Chinese Medicine, Guangzhou, Guangdong, China

**Keywords:** autism spectrum disorder, gut bacteria, immune-inflammation, maternal diet, microbial-gut-brain axis

## Abstract

Autism spectrum disorder (ASD) is a neurodevelopmental disorder that manifests in early childhood, with its specific causes and pathogenesis remaining incompletely understood. The gut bacteria plays a pivotal role in host health and neurodevelopment. Maternal eating disorders may disrupt maternal gut bacteria and subsequently influence fetal and neonatal gut bacteria through the gut-placental axis and breastfeeding. This disruption can ultimately impact the microbial-gut-brain axis, the immune system, neurotransmitter dysregulation, and metabolite abnormalities, thereby increasing the risk of ASD in offspring. This paper reviews the adverse effects of bad maternal dietary habits, including high-sugar, high-salt, high-fat diets, alcohol consumption, dietary fiber deficiency, and the intake of ultra-processed foods, on the gut bacteria. It also explores the mechanisms by which gut microbiota disorder may induce ASD through the immune system, neurotransmitters, and metabolites. Additionally, the article proposes potential strategies to prevent ASD by adjusting dietary structures and enhancing gut bacteria health.

## 1 Introduction

Gut bacteria typically coexist harmoniously with their hosts, contributing significantly to the individual’s health and well-being. The normal gut microbiota constitutes a dynamic ecosystem comprising trillions of bacteria that enhance intestinal mucosal integrity, supply essential nutrients such as vitamins and enzymes, protect the body from pathogens, and actively participate in both the innate and adaptive immune systems (Wang J. et al., 2024). Additionally, gut bacteria are crucial for the cognitive and behavioral functions of the host; disruptions in their balance can jeopardize this symbiotic relationship and pose potential risks for neurodevelopmental deficits (Ahrens et al., 2024).

The dietary habits of the host exert a regulatory effect on gut bacteria, and alterations in these bacteria can significantly influence human health. Poor dietary practices of the mother may have lasting consequences on the brain structure and function of her offspring, potentially leading to neurodevelopmental disorders (Vuong et al., 2020). The fetal gut is not a sterile environment; rather, it possesses a low abundance yet metabolically rich microbiome (Liu X. et al., 2022). There exists a correlation between the mother’s diet and the bacteria present in both the fetal and neonatal gut. Furthermore, eating disorders during pregnancy can adversely affect the mother’s gut microbiota, which subsequently impacts the fetal gut bacteria via the gut-placental axis (Zha et al., 2024). Following birth, eating disorders in breastfeeding mothers may alter the composition of breast milk, thereby influencing the gut bacteria of breastfed infants (Savage et al., 2018).

Autism spectrum disorder (ASD), a neurodevelopmental disorder, is clinically characterized by deficits in language expression, cognitive dysfunction, poor social skills, social isolation, restricted interests, and stereotyped behaviors. It is often regarded as being closely linked to genetic, environmental, immune, inflammatory, and neural pathways (Chen et al., 2024). The composition of gut bacteria in individuals with ASD differs significantly from that of normal individuals, exhibiting decreased microbial diversity and abnormal abundances of specific bacterial populations (Li et al., 2023). Gastrointestinal dysfunction, which includes altered bowel function, abdominal pain, diarrhea, reflux, and vomiting, is observed in approximately 40% of patients with ASD (Varley and Browning, 2024). Moreover, the correlation between gastrointestinal symptoms and the severity of ASD underscores the significance of the relationship between gut microbiota and brain function (Brito et al., 2024). This dysbiosis of gut bacteria may result in abnormal immune system activation, neurotransmitter imbalances, and heightened inflammatory responses (Naufel et al., 2023), potentially influencing the onset and progression of ASD.

**FIGURE 1:**
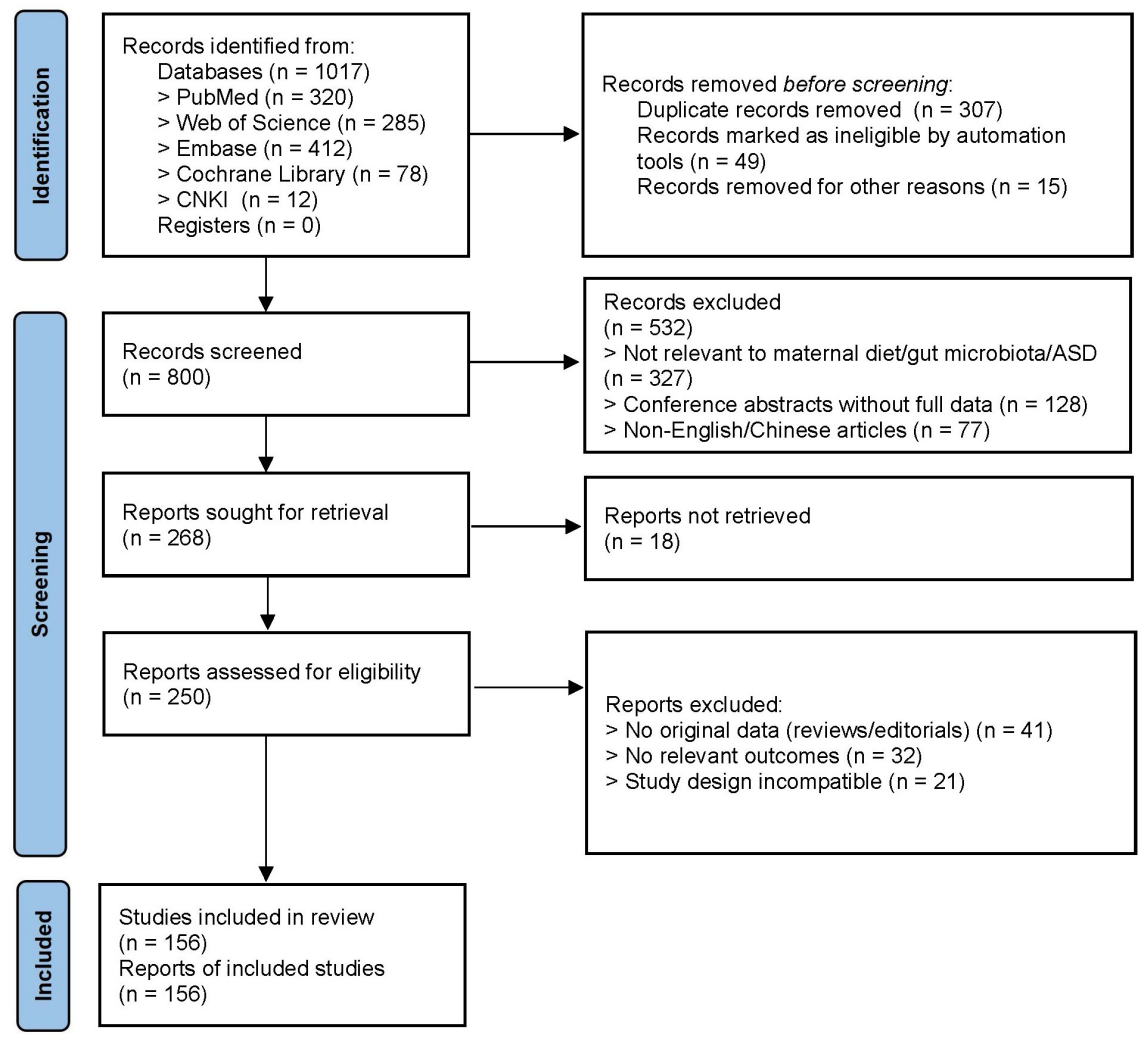
PRISMA flowchart.

This review will explore the potential associations and mechanisms linking maternal diet and gut microbiota to the development of ASD in offspring. The findings aim to assist mothers in minimizing the risk of ASD in their children and to offer more effective strategies for future interventions (Figure 1).

## 2 Maternal eating disorders induce changes in gut bacteria

The gut microbiota comprises approximately 500–1,000 distinct species that play a vital role in maintaining health and influencing various physiological processes. When the ecological balance of this flora is intact, normal physiological functions of the host—such as metabolism, immunomodulation, and nutrient absorption—can be sustained. However, an imbalance in the microbiota may lead to the development of various diseases. Dietary habits are among the most critical factors affecting the gut microbiome and are also a common cause of microbiota imbalance. Unhealthy dietary habits, such as a high-fat diet, can diminish the diversity of the gut microbiota and increase intestinal permeability, allowing harmful substances to enter the bloodstream and trigger systemic diseases (Tomas et al., 2016; Overbeeke et al., 2022). The effects of eating disorders on gut bacteria are illustrated in Table 1.

**TABLE 1:** Effects of eating disorders on gut bacteria.

Eating disorders	Increase	Decrease	Consequences
High sugar intake	*Escherichia* spp., *Staphylococcus* spp.	*Bifidobacterium* spp.	Reduction of beneficial bacteria, Increase of harmful bacteria, Decreased microbial diversity, Inflammation, Intestinal barrier damage
High salt intake	Proteobacteria, Lachnospiraceae, *Bacteroides* spp., *Clostridium* spp., Bacillota/Bacteroidota	*Lactobacillus* spp., *Bifidobacterium* spp., Bacillus fragilis	
High fat intake	Lachnospiraceae, *Bacteroides* spp., *Blautia* spp., Bacillota/Bacteroidota	*Akkermansia* spp., *Faecalibaculum* spp., *Allobaculum* spp., *Prevotella* spp., Bacillus spp. (female)	
Alcohol consumption	Proteobacteria, *Sutterella* spp., *Holdemania* spp., *Clostridium* spp.	*Faecalibacterium* spp.	
Low dietary fiber intake	Bacillota/Bacteroidota		
Ultra-processed food intake	*Enterobacter* spp., *Clostridium* spp.	*Bifidobacterium* spp., Ruminococcaceae spp.	

### 2.1 High sugar intake

High-sugar diet may lead to an imbalance of gut bacteria, promoting the growth of harmful bacteria while inhibiting the growth of beneficial bacteria. This dysbiosis can result in immune dysregulation, trigger inflammatory responses, and damage the intestinal mucosal barrier, ultimately jeopardizing intestinal health (Ki et al., 2024; Castor et al., 2024). Studies have demonstrated that a high-sugar diet induces the overgrowth of harmful bacteria, such as *Escherichia* spp. and *Staphylococcus* spp., while decreasing the abundance of *Bifidobacterium* spp. Additionally, it disrupts the protective mucus layer of the intestinal tract and increases the risk of colitis in mice (Zhang C. et al., 2024). High-sugar diets also lead to a loss of beneficial bacteria in the gut of housefly larvae, thereby reducing their developmental success. However, enhancing rearing substrate with *Lactobacillus fermentans* and *Lactobacillus plantarum* strains isolated from normal housefly larvae not only mitigated the adverse effects of a high-sugar diet on larval development but also increased the diversity of their intestinal microbiota (Voulgari-Kokota et al., 2024). Furthermore, mulberry leaf flower tea has been shown to alleviate the gut bacterial imbalance induced by hyperglycemic rats, promoting the growth of *Bifidobacterium* spp. and *Lactobacillus* spp. while inhibiting the colonization of *Brucella*, *Klebsiella*, *Helicobacter pylori*, and *Alistipes*. This intervention, in turn, reduces oxidative stress and inflammatory responses, as well as lowers blood glucose levels in diabetic rats (Liu C. et al., 2023). Currently, probiotics exhibit potential therapeutic effects in restoring the gut bacterial imbalance caused by a high-sugar diet; however, their specific therapeutic efficacy requires further validation through additional studies and clinical trials.

### 2.2 High salt intake

High-salt diet may disrupt local immune homeostasis in the gut and alter the composition and function of the gut microbiota (Li et al., 2019). Perinatal exposure to a high-salt diet results in increased bacterial abundance of Proteobacteria and *Bacteroides* spp. in the offspring of weanling mice, leading to gut bacteria dysbiosis (Guo et al., 2021). Furthermore, a high-salt diet is associated with a decrease in beneficial bacteria, such as *Lactobacillus* spp. and *Bifidobacterium* spp., while promoting the growth of potentially harmful bacteria, such as *Clostridium* spp. (Dong et al., 2020; Hamad et al., 2022). Research has shown that a high-salt diet decreases the levels of Bacillus fragile and arachidonic acid in the guts of experimental rats, increases the production of corticosterone from intestinal sources, and elevates the levels of corticosterone in both serum and intestines, thereby affecting the normal functioning of gut bacteria (Yan et al., 2020). Additionally, a high-salt diet induces significant changes in serum short-chain fatty acids (SCFAs) and the gut microbiome, decreasing the ratio of Bacteroidota to Bacillota and the relative abundance of *Lactobacillus* spp. in the intestinal tract, which increases the risk of disease in mice (Musiol et al., 2024). In animal studies, high-salt diets have been shown to exacerbate diseases such as colitis, characterized by elevated levels of pro-inflammatory cytokines and a higher frequency of IL-17A-producing cells in the intestinal lamina propria of mice on high-salt diets (Dong et al., 2020; Na et al., 2021). Notably, extracts from green tea and oolong tea, which possess different phytochemical compositions, have been found to improve gut bacteria in rats fed a high-salt diet (Ye et al., 2022). Moreover, a probiotic known as *Lactobacillus plantarum* ZDY2013 has been shown to enhance intestinal barrier function and limit the inflammatory response by increasing the levels of *Lactobacillus* spp. and *Bifidobacterium* spp. While (Wan et al., 2021). These findings provide valuable insights for reestablishing the imbalance of gut bacteria induced by a high-salt diet.

### 2.3 High fat intake

High-fat diet may promote the growth of harmful bacteria, such as *Enterococcus* spp., in the gut, leading to an inflammatory response in the intestinal mucosa (Tan et al., 2021), an imbalance in immune regulation, and alterations in the metabolites of gut microorganisms (Zheng et al., 2024), which trigger a disruption of the gut bacteria. Following high-fat dietary intervention, the composition of gut bacteria in mice was significantly altered, with reductions in strains of *Akkermansia* spp., *Faecalibaculum* spp., and *Allobaculum* spp., while Lachnospiraceae and *Bacteroides* spp. were significantly increased (Duan et al., 2024). Additionally, high-fat diet increases the ratio of Bacillota to Bacteroidota and the relative abundance of *Blautia* spp., while decreasing *Prevotella* spp. and gut microbial alpha diversity (de Noronha et al., 2024). Notably, there were sex differences in gut bacteria dysbiosis induced by a high-fat diet in mice; the relative abundance of Bacillaceae spp. in the fecal bacteria of female mice was reduced, whereas Bacteroidetes were increased in males, suggesting differential responses to a high-fat diet between sexes (Lefebvre et al., 2024). Furthermore, a high-fat diet impairs the intestinal barrier through the production of gut microbiota-derived reactive oxygen species, which induce mitochondrial dysfunction and apoptosis of intestinal epithelial cells (Zeng et al., 2024). Extracellular reactive oxygen species derived from gut bacteria play a crucial role in high-fat diet-induced intestinal barrier disruption and may represent a potential therapeutic target for metabolic diseases associated with high-fat diets. It has been suggested that dietary supplements containing beneficial microorganisms may mitigate the deleterious effects of high-fat diets by modulating the gut microbiome, metabolic pathways, and metabolites. For instance, *Lactobacillus fermentum* HNU312 has been shown to reduce body weight, lipid levels, fat accumulation, and chronic inflammation induced by a high-fat diet through modulation of gut bacteria, enhancement of lipid metabolic pathways, and increase in SCFAs (Li et al., 2024).

### 2.4 Alcohol consumption

Excessive alcohol intake may negatively impact gut bacteria, leading to micronutrient deficiencies, alterations in intestinal epithelial integrity, and increased intestinal permeability (Pohl et al., 2021). Chronic alcohol consumption modifies the diversity and composition of the gut microbiota. The intestinal epithelial barrier comprises a physical barrier formed by a single layer of intestinal epithelial cells and a chemical barrier containing mucus-trapping host regulatory factors and intestinal commensal bacteria. Excessive alcohol consumption directly disrupts both the physical and chemical barriers of the intestinal epithelium, thereby impairing its regulatory capacity (Kuo et al., 2024). Studies indicate that individuals with a history of excessive alcohol consumption exhibit a higher relative abundance of Proteobacteria and a lower relative abundance of beneficial bacteria, such as *Faecalibacterium* spp. Additionally, elevated concentrations of genera such as *Sutterella* spp., *Holdemania* spp., and *Clostridium* spp. are observed in populations characterized by alcohol overconsumption, whereas the production of butyric acid, a gut-healthy SCFA, is significantly reduced in these populations (Bjørkhaug et al., 2019). Certain components of alcohol induce changes in the gut microbiota, leading to increased intestinal permeability and the translocation of endotoxins into the bloodstream. These endotoxins are associated with various diseases, including autoimmune disorders, neurological disorders, and metabolic conditions such as diabetes and cardiovascular disease (Jaquez-Durán and Arellano-Ortiz, 2024). Notably, ceftriaxone has been shown to reduce alcohol intake and partially reverse alcohol-induced ecological dysregulation in rats, significantly mitigating enterococcus-induced impairment of microbial community diversity (Duclot et al., 2024). Substances such as inulin, a type of dietary fiber, have been investigated for their capacity to modulate the composition of gut microbiota. In studies involving patients with alcohol use disorders, inulin supplementation resulted in alterations to the gut microbiota; however, it did not lead to significant improvements in liver function or inflammatory markers when compared to placebo (Amadieu et al., 2022). Notably, inulin demonstrated potential to enhance socialization and increase serum levels of brain-derived neurotrophic factor, indicating a positive effect on gut-brain interactions. Furthermore, certain natural products targeting alcohol may hold promise for applications in promoting food safety and human health, underscoring the need for further research and development to fully harness the potential benefits of these substances (Zhao et al., 2024).

### 2.5 Low dietary fiber intake

Dietary fiber is an essential nutrient in the human diet, primarily derived from unrefined foods such as grains, legumes, vegetables, and fruits, and is vital for overall health. It plays a significant role in gastrointestinal health by regulating the gut microbiota (Guan et al., 2021). Specifically, dietary fiber enhances the activity of enzymes in the gut microbiome that are responsible for carbohydrate degradation, promotes diversity within the gut microbiota, and reduces inflammatory markers (Wastyk et al., 2021). A deficiency of fiber in the diet has been linked to various health issues, including constipation, irritable bowel syndrome, allergies, and immune-related disorders (Hojsak et al., 2022). Research indicates that fiber deficiency alters the metabolome of intestinal microorganisms, distorts the intestinal immune response to inflammation, and modifies gene expression in intestinal epithelial cells, leading to increased susceptibility in experimental mice to colitis induction. Furthermore, increased dietary fiber intake decreases the infiltration of CD3 + , CD4 + , and CD8 + T-lymphocytes, modulates the structure of gut bacteria, and enhances the ratio of Bacillota to Bacteroidota (Li et al., 2021). Additionally, a lack of fiber and SCFAs has been shown to accelerate memory deficits in experimental mice; supplementation with dietary fiber and microbiota metabolite receptors has been found to improve cognition and alleviate memory deficits and neurological damage in a 5xFAD mouse model of Alzheimer’s disease (Zhou et al., 2023). High maternal dietary fiber intake during pregnancy is associated with a reduced risk of obesity and diabetes, as well as a decrease in disease risk factors in offspring (Basu et al., 2021). Maternal consumption of inulin during pregnancy can alter the gut microbiome profile and increase levels of SCFAs, such as propionate and butyrate, in the colon, thereby enhancing fetal development (Peng et al., 2022). Consequently, adequate dietary fiber intake during pregnancy is essential for both maternal and fetal health.

### 2.6 Ultra-processed food intake

Modern ultra-processed foods are characterized by high levels of saturated fats, trans fats, added sugars, salt, and food additives, which can significantly impact gut and overall body health. Furthermore, certain food additives have been linked to metabolic and inflammatory diseases. For instance, additives such as emulsifiers, artificial sweeteners, colorants, and preservatives interact with the gut microbiota, leading to alterations in the gut barrier, the activation of chronic inflammation, and the triggering of aberrant immune responses (Raoul et al., 2022). These additives may also have potentially harmful effects on brain health (Song et al., 2023). Research indicates that children consuming processed foods exhibit a significantly higher abundance of harmful bacteria, such as *Enterobacter* spp. and *Clostridium* spp., alongside a significantly lower abundance of beneficial bacteria, including *Bifidobacterium* spp. and *Ruminococcus* spp. Additionally, these children demonstrate lower fecal concentrations of acetate and butyrate compared to those following a healthy diet, which disrupts intestinal microbial homeostasis and may pose a risk factor for psycho-behavioral disorders in children (Jung et al., 2022). A high intake of ultra-processed foods is associated with lower dietary quality, which may adversely affect maternal health and neonatal outcomes (Ben-Avraham et al., 2023). One study has reported an adverse association between maternal consumption of ultra-processed foods during pregnancy and early childhood language function (Puig-Vallverdú et al., 2022), a critical cognitive aspect of neurodevelopment.

## 3 Effects of changes in maternal gut bacteria on fetal and neonatal gut bacteria

**FIGURE 2:**
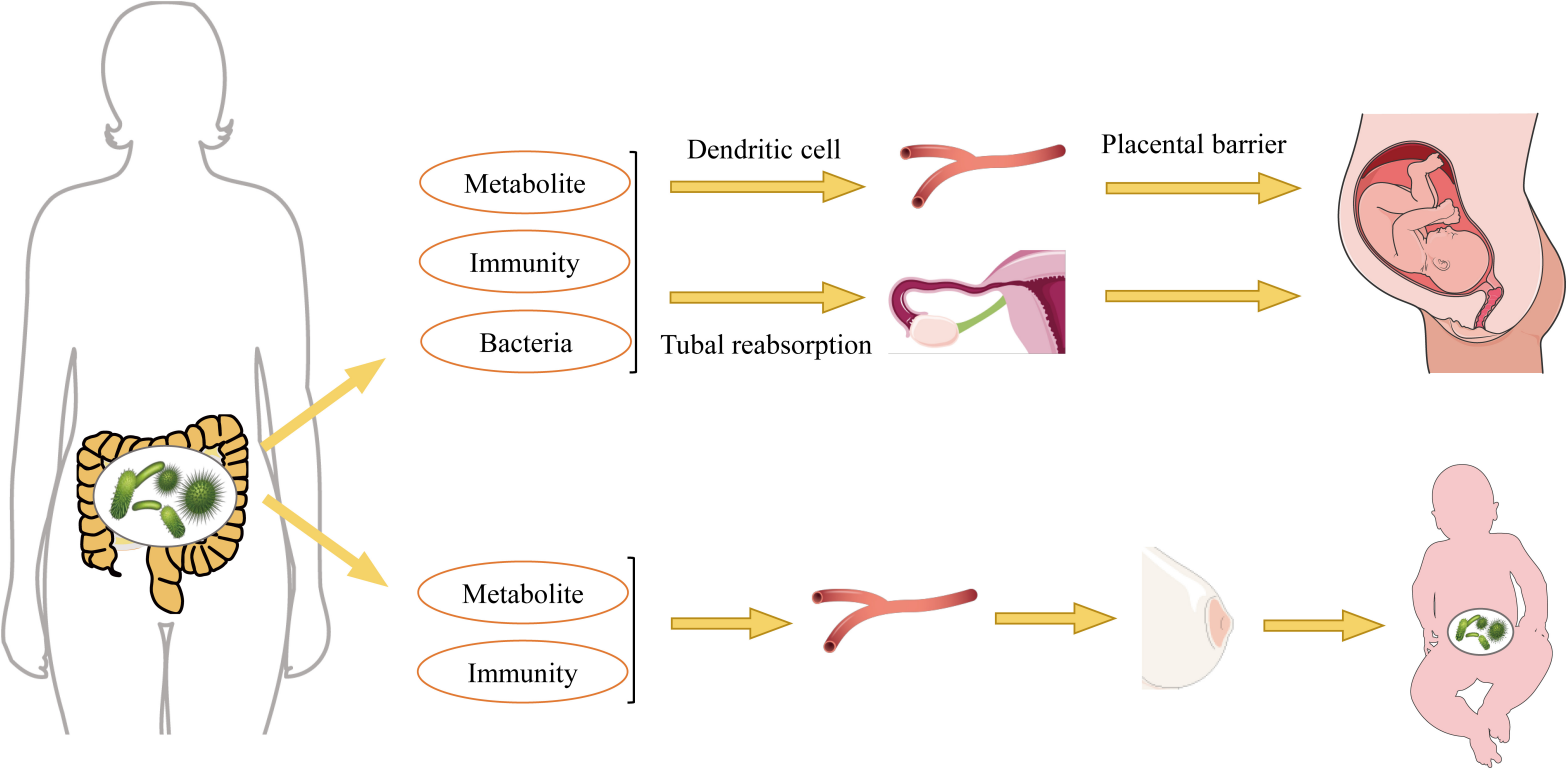
Pathways by which maternal gut bacteria influence offspring gut bacteria.

The maternal microbiome is essential for the healthy growth and development of offspring, exerting long-term effects during fetal growth, with male fetuses potentially being more susceptible to microbial regulation (Husso et al., 2023). Research indicates that the maternal gut microbiome begins to influence fetal health and development during pregnancy, significantly affecting the expression of genes critical for the immune system, neurophysiology, translation, and energy metabolism, all of which are strongly influenced by maternal microbial status prior to birth (Tao et al., 2023). Therefore, alterations in maternal gut bacteria play a crucial role in establishing the gut microbiota in offspring (Figure 2).

### 3.1 Maternal effects on fetal gut bacteria during pregnancy

During pregnancy, significant alterations in the maternal gut microbiota can profoundly influence the development of the fetal microbiota. Bacterial components and products from the maternal microbiome may reach the fetus through transmission via the maternal vascular supply. Observations of microbial DNA translocation from the maternal to the fetal gut suggest that the fetal gut may not be as completely sterile as previously thought. Rather, it may harbor microbial signatures akin to those found in the placenta and maternal gut, indicating that a form of microbial translocation could prime the fetal immune system for the postnatal period (Martinez et al., 2018; Hattoufi et al., 2024). Furthermore, it has been proposed that *in utero* microbes affect the establishment of fetal gut bacteria through translocation, filtering of maternal gut microbes and their metabolites into the peritoneal cavity via the intestinal vasculature, reabsorption through the fallopian tubes, and the transport of blood-borne substances to the uterus facilitated by dendritic cells and leukocytes (Pessa-Morikawa et al., 2022; Dera et al., 2024). The maternal gut microbiota experiences significant changes throughout pregnancy, characterized by alterations in bacterial diversity and composition. For instance, the Lachnospiraceae FCS020 group and Ruminococcaceae UCG-003 bacteria may proliferate during the second trimester, producing essential nutrients such as docosatrienoic acid for fetal growth (Pan et al., 2024). Increased dietary fiber intake enhances the abundance of beneficial bacteria, such as Christobacteriaceae and Fusobacteria, in the mother, which in turn promotes the growth and development of the infant’s gut microbiota (García-Mantrana et al., 2020). Additionally, maternal intestinal *Bifidobacterium* spp. have been shown to support placental morphogenesis, nutrient transport, and fetal growth in mice (Lopez-Tello et al., 2022). Extracellular vesicles derived from maternal gut microbiota serve as a mechanism for the interaction between maternal microbiota and the fetus, playing a crucial role in initiating the prenatal immune system and facilitating fetal gut microbial colonization after birth (Kaisanlahti et al., 2023).

Fetal growth restriction is a complex obstetric complication. A significant reduction in the abundance of the nitrogen metabolism pathway and an increase in the amoebiasis pathway were observed in mothers with fetal growth restriction, indicating that the development of this condition is associated with alterations in the gut microbiota of pregnant women (He X. et al., 2023). In pregnant rats, gut bacteria can mitigate fetal growth restriction by inhibiting the TLR9/MyD88 pathway (Tang et al., 2023). Additionally, the gut microbiota contributes to bisphenol A induced maternal intestinal and placental apoptosis, oxidative stress, and fetal growth restriction by modulating the gut-placental axis (Zhang H. et al., 2024). Treatment with antibiotics in pregnant mice resulted in a significant reduction in gut microbiota diversity, characterized by a decrease in beneficial bacteria such as *Lactobacillus* spp. and an increase in potentially harmful bacteria such as *Clostridium* spp. (Tochitani et al., 2016). Furthermore, the maternal microbiota can influence offspring development through the transfer of microbial metabolites to the fetus. A deficiency in these metabolites can lead to a lack of essential signaling molecules necessary for the development of the fetal immune system. It is well established that the maternal gut microbiota normally produces metabolites, such as SCFAs and other microbial-derived compounds, which play critical roles in regulating immune responses and maintaining gut homeostasis. Under sterile conditions, a deficiency of these metabolites results in significant alterations in the metabolic pattern of the fetal gut, consequently increasing susceptibility to infection and inflammation later in life (Husso et al., 2023). Research has demonstrated that melatonin supplementation restores the intestinal microbiota in heat-stressed pregnant mice by reducing the production of lipopolysaccharides (LPS) and enhancing the proliferation of beneficial microbial communities. This restoration is associated with a decrease in LPS within the maternal gut-placental-fetal axis, which is further linked to improved integrity of both the intestinal and placental barriers. These effects collectively contribute to the protection of the fetus from oxidative stress and inflammatory damage (Wu et al., 2024).

### 3.2 Effect of the period of breastfeeding on the gut bacteria of newborns

Maternal dietary structure can lead to an imbalance in maternal gut bacteria, which subsequently affects the levels of probiotics and prebiotics in breast milk, thereby influencing the healthy development of the infant’s gut bacteria (Catassi et al., 2024). Breast milk not only provides essential nutrition for growing infants but also serves as a source of commensal bacteria that can enhance infant health by preventing pathogen adherence and promoting the intestinal colonization of beneficial microorganisms (Lyons et al., 2020). The pediatric microbiome, encompassing both gut and skin communities, is shaped by various factors, with breastfeeding being one of the most significant (Golebiewski et al., 2021). Different bioactive components of breast milk, including oligosaccharides, lactoferrin, and secretory immunoglobulins, modify the composition of the neonatal microbiota. Additionally, breast milk contains numerous factors that are associated with the maturation of the infant’s immune system and the development of gut microbiota (Moossavi et al., 2019). It has been observed that simple carbohydrates consumed by mothers positively correlate with the presence of Enterobacteriaceae in breast milk. In contrast, there is a negative correlation between simple carbohydrate intake and beneficial bacteria such as *Bifidobacterium* spp. (Londoño-Sierra et al., 2023). This suggests that an unbalanced diet, particularly one high in simple carbohydrates, may lead to a less favorable composition of the microbiota in breast milk, potentially adversely affecting the infant’s gut microbiota. The serine and glycine content of mare’s breast milk is positively correlated with the shaping of the foal’s gut microflora, particularly Bacteroidaceae (Mady et al., 2024). Notably, the absence of *Bifidobacterium* spp., especially the depletion of genes required for the utilization of breast milk oligosaccharides from the macrogenome, can lead to systemic inflammation and immune dysregulation early in life (Henrick et al., 2021). Therefore, if exclusive breastfeeding is not feasible, it is essential to adjust the composition of infant formulas to include specific HMO-2’-fucose-based lactose, either alone or supplemented with lactulose-n-neotetrasaccharide. This adjustment can stimulate the development of gut bacteria, predominantly *Bifidobacterium* spp., balance the gut microbiota, and enhance the immunity of newborns (Vandenplas et al., 2020). Maternal probiotic supplements, including *Lactobacillus* spp., *Bifidobacterium* spp., *Streptococcus thermophilus*, and *Streptococcus brassica*, are effective in harmonizing the breast milk and infant gut microbiomes to promote infant health, offering a wide range of clinical benefits and safety (Alemu et al., 2023). The composition of breast milk is dynamic and can vary according to the mother’s nutritional intake. The consumption of specific nutrients, such as polyunsaturated fatty acids and vitamins, has been shown to correlate with the diversity of the breast milk microbiota, which is advantageous for the establishment of infant gut bacteria (Padilha et al., 2019). This indicates that a balanced maternal diet rich in essential nutrients is crucial for promoting a healthy microbiota in breast milk, thereby supporting infant gut health.

Notably, when exclusive breastfeeding is not feasible, compositional differences in infant formula significantly impact the colonization and development of the neonatal gut microbiota (Zhang et al., 2025). Studies have shown that formula-fed neonates exhibit a significantly reduced abundance of *Bifidobacterium* spp. and a corresponding increase in the proportion of Enterobacteriaceae within the gut. This microbial dysbiosis may lead to impaired intestinal barrier function, thereby increasing the risk of systemic inflammation and neurodevelopmental disorders (Charton et al., 2022). To bridge the gap between formula and breast milk, researchers have begun incorporating specific human milk oligosaccharides (HMOs), such as 2’-fucosyllactose and 3-fucosyllactose, into formulas. These oligosaccharides not only promote *Bifidobacterium* spp. colonization but also improve immune responses and enhance intestinal barrier function (Bénet et al., 2024), consequently increasing gut microbial diversity (Catassi et al., 2024). Furthermore, compared to standard cow’s milk-based infant formula, goat milk-based formula selectively increases the relative abundance of beneficial bacteria such as *Blautia* spp., *Roseburia* spp., *Alistipes* spp., and *Muribaculum* spp., facilitating the establishment of a favorable microbial environment and accelerating metabolism in infants (Chen et al., 2023). Additionally, infant formula supplemented with probiotics helps optimize the intestinal environment, modulate microbial composition, and enhance microbiota metabolic activity (Eor et al., 2023).

## 4 Mechanisms of ASD induction by gut bacteria disorders

**FIGURE 3:**
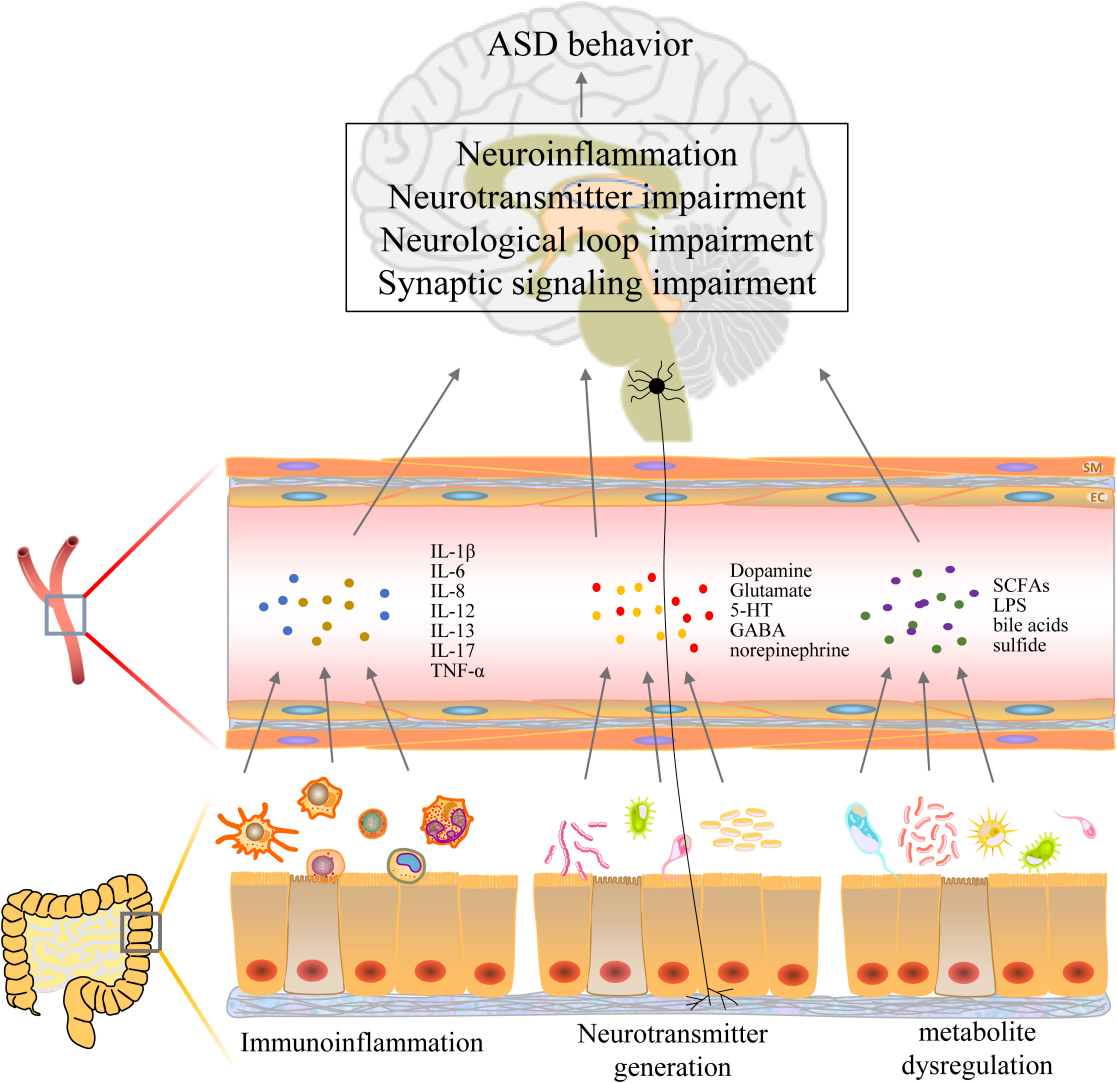
Mechanisms of autism spectrum disorder (ASD) induction by gut bacteria via the gut-brain axis.

The gut microbiota has been linked to early life development, influencing fetal and infant brain development and behavior (Table 2). Research indicates complex interactions between gut bacteria and the brain that affect cognitive function, emotional states, and neurological health through information transfer and regulation via the gut-brain axis. The gut microbiota and its metabolites are regulated by what is known as the microbiota-gut-brain axis (MGBA), which features a bidirectional communication system that governs neurodevelopment and cognitive function (Khan et al., 2024). The MGBA impacts brain development and function through mechanisms involving the hypothalamo-pituitary-adrenal (HPA) axis, immune signaling, and the dysregulation of neurotransmitters or inflammation, as well as through microbial metabolites such as short-chain fatty acids, tryptophan derivatives, and abnormalities in bile acids (BA) that influence brain development and function (Figure 3). Research suggests that alterations in the gut microbiome may be associated with ASD. A meta-analysis revealed that children with ASD are more likely to experience gastrointestinal disorders than neurologically typical children, particularly functional constipation. This finding underscores the importance of screening for gastrointestinal problems within this population, suggesting a significant relationship between gut health and behavioral symptoms (Luna et al., 2016; Carmel et al., 2023). Additionally, reduced levels of *Bifidobacterium* spp., acetate, propionate, and butyrate are commonly observed in the gut microbiota profiles of patients with ASD, alongside diminished levels of *Bacteroides* spp. and other states of ecological dysbiosis (Dargenio et al., 2023). Sex differences are evident at all levels of the MGBA, with sex steroids influencing the composition of the gut microbiota. These microbes, in turn, can modulate the levels of biologically active sex steroids, as well as hormones and microbes that interact with enteroendocrine cells, thereby affecting downstream activity in the enteric nervous system, vagus nerve, and brain (Hokanson et al., 2024). Research has shown that genera such as *Streptococcus* spp., *Bifidobacterium* spp., *Clostridium* spp., *Bacteroides* spp., and *Blautia* spp. can influence the MGBA network during exercise (Xue et al., 2023), suggesting a potential mechanism for enhancing microbiota targeted at ASD.

**TABLE 2:** Literature review table on gut microbiota and ASD.

Author (year)	Study aim	Geographical area	Sample size	Key results
Kang et al., 2017	To evaluate the effects of microbial transplantation therapy on gut microbiota, gastrointestinal symptoms, and behavioral symptoms in children with ASD	America	Treatment group: 18 children with ASD (7–16 years) Control group: 20 neurotypical children	Gastrointestinal symptoms improved by 80%, significant enhancement in ASD behavioral scores, increased gut microbiota diversity, colonization of *Bifidobacterium* and *Prevotella* increased, and the virome shifted toward the donor type.
Wan et al., 2022	Identification of gut microbiota biomarkers for ASD and exploration of microbial developmental trajectories	China	146 children (72 with ASD vs. 74 Typically developing (TD) children)	Five bacterial species were identified that can distinguish children with ASD from TD children. Children with ASD exhibit a significant lag in the maturation of their gut microbiota compared to their age-matched TD peers. The ASD group showed a marked reduction in the activity of neurotransmitter synthesis pathways (tryptophan/glycine). An enrichment of potentially pathogenic bacteria such as *Clostridium* was observed in the ASD group, alongside a deficiency in beneficial bacteria like *Faecalibacterium*.
Liu G. et al., 2022	Investigation of the impact of early-life gut dysbiosis on hippocampal function and behavioral outcomes	China	3∼10 mice/group.	Gut dysbiosis leads to the occurrence of anxiety, decreased spatial memory, and social deficits, with reduced neurogenesis and synaptic plasticity, and elevated expression of inflammatory genes. Elevated serum levels of 4-methylphenol induce ASD-like behaviors and neuronal apoptosis. Fecal microbiota transplantation reverses behavioral and hippocampal impairments.
Kang et al., 2020	Analysis of differences in plasma/fecal metabolites between children with ASD and TD peers, and exploration of metabolite regulation following microbiota transplantation therapy (MTT).	America	18 ASD (with chronic gastrointestinal (GI) symptoms) 20 TD	Serum levels of ten metabolites were significantly altered in the ASD group, and following MTT treatment, eight of these metabolites showed marked improvement (approaching TD levels). Among them, p-cresol sulfate was negatively correlated with the abundance of Desulfovibrio, IMP was negatively associated with the improvement of GI symptoms, and elevated methylsuccinic acid was positively correlated with behavioral improvement.
Chen et al., 2020	Investigation of shared gut bacteria between children with ASD and their mothers and associations with developmental levels and social deficits	China	ASD group: 76 children and their mothers TD group: 47 children and their mothers	Children with ASD exhibit distinct gut microbiota compared to typically developing children, with a positive correlation between the abundance of CAG15 and developmental levels. Children with ASD share specific bacteria from their mothers (such as Lachnospiraceae ASV3491/790), and those sharing specific Veillonella/Coprococcus types of the gut microbiota exhibit milder social deficits in ASD.
De Sales-Millán et al., 2024	Comparison of gut microbiota composition and functional metabolism between children with ASD and neurotypical children, exploring the significance of gender differences and metabolic dysregulation	Mexico	ASD: 30 individuals (25 males/5 females) neurotypical children: 31 individuals (26 males/5 females)	In the ASD group, there was an increase in *Prevotella* and *Clostridium* XI, and a decrease in *Blautia* and *Clostridium* XVIII. The ASD female group uniquely had an increase in Megamonas. Oscillibacter and Acidaminococcus showed gender differences, with an increase in *Lactobacillus* observed only in individuals with severe ASD. The ASD group exhibited enrichment in the amine/amino acid degradation pathway and a reduction in the carbohydrate fermentation pathway.
Wan et al., 2024	Exploring the interactions between the virome and bacteriome in the gut of children with ASD and their impact on neuroactive metabolism	China	ASD group: 60 TD group: 64	In the gut of children with ASD, an enrichment of phages targeting *Clostridium*, Bacillus, and Enterobacteria was observed, accompanied by a reduction in viral diversity. The stability of the bacterium-virus interaction network was compromised, and the phage-mediated capacity to encode pathways for the synthesis of neuroactive amino acids (such as glycine and valine) was significantly diminished.
Cai et al., 2025	To explore the common characteristics and diagnostic value of intestinal microorganisms in children with ASD and ADHD	China	ASD:113 ADHD: 43 ASD + ADHD: 8 Health control: 120 (2–11 years old)	*Bifidobacterium* in the intestine of children with ASD/ADHD increased significantly, while beneficial bacteria such as *Bacteroides* and *Faecalibacterium* decreased, and the flora network structure was simpler. The model based on the characteristics of flora can effectively distinguish patients from healthy children, and found 4899 disordered microbial metabolic functions (such as decreased synthesis of neuroactive substances).
Turriziani et al., 2022	To evaluate the effect of PEG therapy on behavioral symptoms of autistic children with chronic constipation and the regulation of urine on cresol	Italy	T0/T1: 21 T2: 17 (2–8 years old)	Gastrointestinal (GI) improvement significantly alleviated hyperactivity (−26.5%), anxiety (−30.9%), social deficits (−20.4%), and stereotyped behaviors (−39.3%). Urinary p-cresol levels showed no consistent trend (increased at Timepoint 1 and decreased at Timepoint 2) and were not significantly correlated with behavioral improvements (with only a marginal correlation observed for anxiety).
Mendive Dubourdieu and Guerendiain, 2023	To analyze the difference of intestinal flora between ASD and TD children and its correlation with dietary intake and nutritional status.	Uruguay	ASD group: 30 TD group: 28	Children with ASD exhibited significantly lower levels of *Bifidobacterium longum* and higher levels of *Clostridium glycolicum* compared to TD children. ASD children on a gluten-free/casein-free diet had significantly lower levels of bifidobacteria than those not on dietary intervention, with dairy intake positively correlated with bifidobacteria levels and gluten-free grain intake positively correlated with *Faecalibacterium*. Overweight children, particularly those with ASD, showed decreased levels of *Roseburia* and *Faecalibacterium prausnitzii*, while Eubacterium ventriosum and Flavonifractor plautii were increased.
Bin-Khattaf et al., 2022	To evaluate the effect of probiotics on GABA/glutamate signal imbalance in propionic acid-induced autism model rats.	Saudi Arabia	40 rats	Probiotics, particularly pure *Bifidobacterium infantis*, significantly reduced oxidative stress and enhanced brain levels of GABA as well as the expression of GABA receptors. Among the probiotics tested, *Bifidobacterium* demonstrated the most pronounced effects, followed by a probiotic mixture, while *Lactobacillus bulgaricus* exhibited relatively weaker efficacy.
Zhang et al., 2018	To analyze the characteristics of intestinal flora of children with ASD in China and its correlation with diseases.	China	35 ASD children 6 TD children	In the ASD group, the ratio of Bacteroidetes to Firmicutes was significantly elevated, with decreased abundance of the genera Streptococcus and Veillonella. Producers of butyrate and lactate were also reduced. A disease network constructed based on microbial community similarity revealed a positive correlation between ASD and periodontal disease, while a negative correlation was observed between ASD and type 1 diabetes.

### 4.1 Immune system disorder

The microbial community exists in symbiosis with the host, contributing to homeostasis and the regulation of immune function (Hou et al., 2022). The intestinal microbiota plays a crucial role in maintaining the integrity and function of the intestinal barrier, thereby supporting homeostatic balance (Di Tommaso et al., 2021). The permeability barrier of the intestinal mucosa protects against nutrient absorption and damage from external factors. This barrier comprises epithelial cells, immune cells, and their secretions. Epithelial cells create chemical and physical barriers that separate the intestinal microbiota from immune cells, while intestinal immune cells are instrumental in sustaining a healthy microbial community and enhancing epithelial barrier function (Kayama et al., 2020). Given that 70%–80% of immune cells reside in the gut (Wiertsema et al., 2021), any disruption of the intestinal barrier may lead to a systemic inflammatory response and tissue damage. Evidence of innate immune dysfunction is frequently observed in children with ASD, often correlating with behavioral deterioration (Hughes et al., 2023). The immune hypothesis is regarded as a significant contributor to the pathogenesis of autism, providing insights into the variations in clinical phenotypes and comorbidities that influence the disease’s course and severity (Robinson-Agramonte et al., 2022). Immune dysfunction in peripheral tissues may be closely linked to the neurodevelopmental deficits seen in ASD.

Dysbiosis of gut bacteria may lead to abnormal alterations in various immune cell types, including macrophages, dendritic cells, T cells, B cells, and NK cells (Yang and Cong, 2021). This disruption can affect the normal functioning of the immune system and increase the risk of disease. Notable differences in the resting states of dendritic cells and monocytes have been observed in patients with ASD compared to healthy individuals (Wei et al., 2022). Additionally, both CD4 + T cells and regulatory B and Treg cells were found to be significantly reduced, while Th17 lymphocytes were increased (De Giacomo et al., 2021; Ellul et al., 2021). Three immune cell types including monocytes, M2 macrophages and activated dendritic cells correlated to varying degrees with 15 identified hub genes associated with ASD (Li et al., 2022). *Bifidobacterium* spp. are known to upregulate suppressor regulatory T cells, maintain intestinal barrier function, modulate the activity of dendritic cells and macrophages, and inhibit intestinal Th2 and Th17 programs, thereby attenuating immune-inflammatory responses (Gavzy et al., 2023). Dysbiosis results in increased secretion of LPS, which, upon binding to endothelial cells via TLR2/4, activates dendritic cells and macrophages. This activation leads to abnormal M1/M2 polarization of macrophages, resulting in heightened inflammatory markers, an imbalance in immune homeostasis, and the potential induction of ASD-like neurodevelopmental disorders (Cristofori et al., 2021; Huang et al., 2021; Takada et al., 2024). Transplantation of fecal microbiota reduces the activation of microglia and astrocytes in the substantia nigra of the mouse brain, as well as the expression of components of the TLR4/TNF-α signaling pathway in both the gut and the brain, thereby inhibiting neuroinflammation (Sun et al., 2018). Recent studies have highlighted NK cell dysfunction in children with ASD, characterized by reduced cytotoxic activity, elevated levels of activation, and an increased number of NK cells in individuals with ASD. These findings suggest dysregulated NK cell activity in the peripheral blood of children diagnosed with ASD (Kaminski et al., 2023).

Expression levels of TNF-α, IL-1β, IL-6, IL-8, and IL-17 were significantly upregulated in the whole blood of patients with ASD compared to healthy subjects (Eftekharian et al., 2018; Ferencova et al., 2023). IL-8 has demonstrated parental cytokine levels in offspring-parental regression analyses and has been associated with deficits in social interaction, cognitive impairments, and motivational imbalances, suggesting it may serve as a potential biomarker for ASD (Shen et al., 2021). Increased levels of IL-1β and IL-17A mRNA expression in colonic tissues were observed in early immune-activated ASD mice (Hoogenraad and Riol-Blanco, 2020). Additionally, elevated levels of IL-6 and IL-1β, along with decreased levels of CD68 and TGF-β mRNA, were noted in the hippocampus and prefrontal cortex of early immune-activated male mice. This may be attributed to gut barrier dysfunction, which increases plasma LPS and lipopolysaccharide-binding protein concentrations, significantly elevating TNF-α, IL-8, IL-12, and IL-13 levels. Furthermore, inflammatory signals are transduced to the brain via TNF receptor superfamily member 1A, leading to a significant increase in microglial cell activation markers and chemokines in the cerebral cortex, ultimately inducing central nervous system disorders (Mitchell et al., 2022; Bundgaard-Nielsen et al., 2023; Yang et al., 2023). The ability to ameliorate intestinal inflammation through the use of probiotics or intestinal mucosal anti-α4β monoclonal antibodies mitigates the neuroinflammatory state, restores synaptic function, enhances spatial learning, and reduces memory deterioration, which in turn improves ASD-like behaviors (Ku et al., 2023; Butera et al., 2024). Additionally, the ketogenic diet decreases the expression levels of TNF-α, IL-1β, and IL-6 in the plasma, prefrontal cortex, and hippocampus of ASD-like mice, alleviates social deficits, repetitive behaviors, and memory impairments, and reduces inflammation and oxidative stress while remodeling the gut-brain axis (Olivito et al., 2023).

In conclusion, dysregulation of gut bacteria may disrupt the balance of immune cells and compromise the stabilization of the immune system. This disruption can lead to immune dysregulation and abnormal development of the nervous system, which are associated with the onset and progression of ASD. Therefore, immunotherapy aimed at suppressing neuroinflammation warrants intensive investigation as a potential avenue for the treatment or intervention of ASD (Shen et al., 2023).

### 4.2 Neurotransmitter disorder

Neurotransmitter function serves as a crucial link in the regulation of the MGBA, and the dysregulation of gut bacteria may impact various neurotransmitters, subsequently affecting the function and regulation of the nervous system (Table 3). The alpha diversity of gut microbiota in children with ASD did not show significant changes with age. However, children with ASD who also had comorbid gastrointestinal disorders exhibited reduced gut bacteria diversity, characterized by a depletion of *Sutterella* spp., *Prevotella* spp., and *Bacteroides* spp. species, alongside associated dysregulation of metabolic activity. Furthermore, the neurotransmitter metabolic network involving dopamine, glutamate, 5-hydroxytryptophan (5-HT), and γ-aminobutyric acid (GABA) differed from that of typically developing children (Dan et al., 2020). Recent pharmacological studies have identified that the walnut-derived peptide LPLLR can maintain gut barrier and blood-brain barrier (BBB) integrity in colitis mice, remodel the abundance of various gut microbiota, increase *Prevotella* spp. associated with tryptophan, 5-HT, and 5-hydroxyindoleacetic acid, and reverse gut bacterial disruption. Additionally, LPLLR has been shown to enhance the learning ability and memory of cognitively impaired mice (Qi et al., 2023). Interventions utilizing probiotics and oligofructose to modulate the MGBA have demonstrated efficacy in alleviating ASD symptoms while reducing the hyper-serotonergic state and dopamine disorders in metabolism (Wang et al., 2020).

**TABLE 3:** Mechanisms of ASD induction by gut bacteria via neurotransmitters.

Neurotransmitters	Related gut bacteria	ASD-like behavior	Mechanisms of ASD induction
5-HT	*Bacteroides* spp., *Clostridium* spp.	Mood disorders, social disorders, repetitive behaviors	Gut bacteria affect 5-HT synthesis through tryptophan metabolism, and abnormal 5-HT levels affect brain development and neural signaling.
GABA	*Lactobacillus* spp., *Bifidobacterium* spp.	Anxiety, hyperventilation, sleep disorders	Gut bacteria affect GABA synthesis through glutamate metabolism, and a decrease in GABA leads to increased neuronal excitability and affects neural development.
Dopamine	*Escherichia coli*, *Enterococcus* spp.	Attention deficit, stereotyped behavior, movement disorders	Gut bacteria affect dopamine synthesis through tyrosine metabolism, and abnormal dopamine levels affect the rewards system and motor control.
Glutamate	*Bacteroides* spp., *Prevotella* spp.	Neuronal hyperexcitability, cognitive impairment	Gut bacteria influence glutamate levels through glutamine metabolism, and glutamate excess leads to neuronal excitotoxicity.

Autism spectrum disorder behavior is believed to result from dysfunction within the midbrain dopaminergic system (Paval, 2023). Defects in the gut microbiota EPHB6, which plays an important role in gut homeostasis, disorganize the gut bacteria and lead to vitamin B6 and dopamine deficiencies, inducing ASD-like behavior in mice (Li et al., 2020). In mice experiencing chronic stress-induced cognitive deficits, significant reductions in 5-HT levels, increased autophagy in the hippocampus, heightened neuroinflammatory responses, and disruption of gut bacteria were observed (Ma et al., 2023). Disruption of gut microbiota homeostasis can modulate tryptophan levels (a precursor of 5-HT) via various metabolic pathways, resulting in dysregulation of 5-HT levels, which may contribute to behavioral symptoms associated with ASD, such as anxiety and social withdrawal (Lim et al., 2017; Chang et al., 2024). Feeding Mucinophilic Fusobacteria to ASD mice restored BDNF and 5-HT expression levels, significantly ameliorating cognitive dysfunction (Kang et al., 2024). Notably, oral administration of 5-HT to neonatal mice promotes early immune system development by facilitating long-term T-cell-specific antigenic immune tolerance to dietary antigens and commensal bacterial-mediated immunity (Sanidad et al., 2024), highlighting the important modulatory role of neurotransmitters on the immune system. GABA has been shown to influence intestinal integrity by regulating intestinal mucins and tight junction proteins, as well as inhibiting vagal signaling (Ikegami et al., 2024). Variations in gut microbiota composition and GABA excretion in infants may affect the risk of developing ASD (Zuffa et al., 2023). GABA supplementation has been found to restore gut bacterial diversity, improve antioxidative stress and inflammatory responses, normalize neurotransmitter levels, and protect the organization of the gut and brain in brain-injured mice (He et al., 2024).

Some gut bacteria are capable of direct metabolic production of neurotransmitters, *Bifidobacterium* spp., *Bacteroides* spp. and *Lactobacillus* spp. are producers of GABA, which helps to regulate neuronal excitability and has been linked to anxiety and mood disorders, and their dysregulation may contribute to the development of neurodevelopmental disorders such as ASD (De-Paula et al., 2018; Vellingiri et al., 2022). Additionally, certain species within the genus Streptococcus produce 5-HT and norepinephrine, both of which are crucial for regulating mood and emotional responses (Reid et al., 2022; Liu Z. et al., 2023). Furthermore, the gut-brain axis functions as a bidirectional communication system, where neurotransmitters produced in the brain can influence gut motility and microbiota composition, thereby creating a feedback loop that may perpetuate dysbiosis. In individuals with ASD, stress and anxiety are often exacerbated, leading to alterations in gut motility and permeability. This can potentially result in further dysbiosis and a worsening of the gastrointestinal symptoms typically associated with ASD (Plaza-Díaz et al., 2019; Dan et al., 2020).

### 4.3 Metabolite disorder

Gut microbial metabolites play a significant role in the mechanisms of the MGBA by influencing gut and brain functions through complex signaling pathways. Normal gut bacteria ferment dietary fiber to produce SCFAs, including propionic, butyric, and acetic acids, which can cross the BBB and exert neuroprotective effects by modulating neuroinflammation and promoting the integrity of the BBB (Horvath et al., 2022; Mann et al., 2024). A decreased abundance of lactobacilli and propionic acid among SCFAs have been observed in the feces of some children with ASD (He J. et al., 2023), suggesting a potential predictor of ASD. Butyric acid, in particular, has been shown to play a crucial role in the development of ASD. Treatment with sodium butyrate in ASD models has been found to modulate the HPA axis and improve anxiety and social deficits in the offspring of LPS-exposed ASD rats (Wang et al., 2023). Additionally, ammonia produced by normal gut microbes contributes to host stress relief, modulates host stress vulnerability, and provides a gut-brain signaling basis for emotional behavior. Notably, fecal ammonia concentrations in children with ASD are significantly higher than those in typically developing children (Wang et al., 2012), and elevated ammonia levels can lead to decreased GABA levels, exacerbating excitatory signaling in the brain (Wang et al., 2019). Furthermore, impairing the function of the BBB allows neurotoxic compounds to enter the brain, leading to neuroinflammation and neuronal damage associated with ASD symptoms (Cuomo et al., 2023).

Certain bacteria in the gut produce LPS, and an imbalance in gut microbiota may lead to an increased release of LPS, subsequently triggering an inflammatory response in the gut. In experimental studies involving mice, the dual induction of antibiotics and LPS resulted in weight loss, fecal abnormalities, disruption of the mucosal structure in the ileum, increased permeability of the intestinal barrier, and structural disruption of the microbiota (Wang Y. et al., 2024). Excessive production of the intestinal metabolite LPS can activate M1 microglia in the brain, leading to decreased dendritic spine density and reduced cognitive function (Peng et al., 2024). Both dietary and microbial factors can influence the metabolism of intestinal BA, resulting in abnormal accumulation or degradation of BA, which in turn affects lipid metabolism and absorption. Furthermore, BA metabolites can regulate T cell homeostasis via intestinal RORγ, with normal metabolism contributing to the stability of the host immune system (Song et al., 2020). Circulating BA can cross the BBB and reach the central nervous system through passive diffusion or BA transporter proteins, exerting neuromodulatory effects. Altered BA metabolites have been identified as potential causative factors for various neurological disorders (Xing et al., 2023). Additionally, certain bacteria produce sulfide in the gut, primarily including Desulfovibrio spp. and Bilophila wadsworthia. At high concentrations, sulfide is cytotoxic and disrupts the integrity of the intestinal epithelium and mucus barrier, triggering intestinal inflammation (Pimenta et al., 2024). However, it has also been suggested that sulfide may confer protective effects on neurons through mechanisms such as antioxidant and anti-inflammatory actions, thereby maintaining normal nervous system function (Gao et al., 2022).

## 5 Discussion

Research indicates that pregnant women with eating disorders face a significantly increased risk of having offspring diagnosed with ASD and Attention-Deficit/Hyperactivity Disorder (Mantel et al., 2022). Maternal dietary disorders may impair gut microbiota, which subsequently affects fetal and neonatal gut bacteria through the gut-placental axis and breastfeeding. This disruption can ultimately influence the MGBA, immune system function, immune responses, neurotransmitter levels, and metabolism, thereby contributing to the onset and progression of ASD. Modifying maternal diet and enhancing gut microbiota health may serve as effective strategies for preventing ASD in children. It is recommended that mothers maintain a balanced diet during pregnancy and breastfeeding, avoiding high-sugar diets, high-salt diets, and high-fat diets, alcohol intake and ultra-processed foods intake, while increasing dietary fiber intake. Probiotic and prebiotic supplementation may assist in restoring gut microbiota balance and improving gut barrier function and immune regulation. However, further large-scale clinical studies are necessary to determine whether maternal probiotic supplementation can effectively reduce the risk of ASD in offspring.

The intestinal microbiota constitutes a dynamically changing ecosystem that significantly influences the cognitive and behavioral functions of the host. Poor maternal dietary habits can lead to a reduction in gut bacterial diversity, the proliferation of harmful bacteria, and a decrease in beneficial bacteria, ultimately affecting the establishment of normal gut bacteria in the offspring. However, maternal genital tract flora can influence the establishment of fetal gut bacteria through upward migration or direct contact during labor, with vaginal and rectal bacterial microbiota converging in the last trimester of gestation and the second month of life, and damage to the genital tract bacteria may be an important cause of fetal gut bacteria damage (Shin et al., 2023). Additionally, damage to maternal gut bacteria may trigger an autoimmune inflammatory response, with immune activation and the transfer of inflammatory factors potentially playing significant roles in increasing the risk of ASD in the offspring (Suprunowicz et al., 2024).

The MGBA plays a significant role in ASD, as the gut microbiota and its metabolites influence cognitive function, emotional state, and neurological health through messaging and regulation via the MGBA axis. The primary mechanisms involved include immune system dysregulation, neurotransmitter abnormalities, and metabolite irregularities. Furthermore, the HPA axis is an integral component of the MGBA that warrants attention. Although direct evidence linking gut microbes to an increased risk of developing ASD through the HPA axis is lacking, pro-inflammatory cytokines induced by gut microbes may activate the HPA axis, potentially leading to neurological disorders (Seguella et al., 2021).

Recent studies have identified significant disruptions in the gut microbiota of children with ASD, encompassing 14 archaeal, 51 bacterial, 7 fungal, and 18 viral species, along with 27 microbial genes and 12 metabolic pathways (Su et al., 2024). Mycobiome analyses further revealed elevated abundances of Ascomycota and *Candida albicans* in the ASD cohort (Retuerto et al., 2024). Notably, modulating gut microbial composition may mitigate Candida overgrowth, thereby alleviating gastrointestinal comorbidities and ASD-related symptoms (Herman and Herman, 2022). Virome investigations highlight ASD-associated shifts in gut viral communities, particularly the enrichment of Clostridium phages, Bacillus phages, and Enterobacteria phages. These phage alterations correlate strongly with ecological dysbiosis in the ASD gut virome (Wan et al., 2024). Critically, disrupted cross-kingdom interactions between the bacteriome and virome in ASD may impair microbial pathways involved in neuroactive metabolite biosynthesis. These findings underscore a compromised bacteriophage-bacterial network in ASD, offering novel insights into the role of phages in ASD pathogenesis and potential therapeutic targets. Further research should explore maternal gut microbiota diversity and its association with offspring ASD risk, with emphasis on non-bacterial components (e.g., archaea, fungi, and viruses) mediated by the MGBA. Such investigations will advance our understanding of microbial ecosystem complexity in ASD and inform future mechanistic and translational studies.

However, although this review summarizes the potential mechanisms by which maternal dietary disorders influence offspring ASD risk via the gut microbiota, direct evidence proving the impact of diet on offspring ASD is still lacking. Future research should focus on two key aspects. First, large-scale prospective cohort studies and rigorous randomized controlled trials are needed to evaluate the clinical efficacy and safety of specific probiotic combinations, dietary fiber fortification, or personalized nutritional interventions during pregnancy/lactation for preventively modulating maternal gut microbiota and improving offspring neurodevelopmental outcomes in ASD. Second, experimental studies are required to deeply elucidate how specific dysregulated maternal gut bacterial species or their metabolites (e.g., SCFAs, neuroactive substances, inflammatory factors) precisely regulate fetal/neonatal neurodevelopmental pathways by crossing the placental barrier or via breast milk, particularly during critical developmental windows. Furthermore, the long-term effects of maternal gut microbiota dysbiosis on offspring gut microbiota colonization and its transgenerational association with ASD phenotypes warrant further investigation. Addressing these key questions will provide a solid scientific foundation and translational pathways for developing novel ASD prevention strategies based on maternal gut microbiome modulation.

## 6 Conclusion

This review systematically elucidates that maternal poor dietary habits (characterized by high-sugar, high-salt, and high-fat diets, alcohol consumption, dietary fiber deficiency, and excessive intake of ultra-processed foods) disrupt maternal gut microbiota homeostasis. This dysbiosis subsequently impacts fetal and neonatal gut microbiota via the “gut-placental axis” during pregnancy and alterations in breast milk composition during lactation, thereby increasing offspring risk of ASD. The core mechanism involves the dysregulated gut microbiota triggering multiple pathological processes through the MGBA. Specifically, microbiota imbalance activates pro-inflammatory responses and disrupts immune homeostasis, inducing neuroinflammation and aberrant brain development. Furthermore, microbiota disruption interferes with the synthesis and regulation of key neurotransmitters (e.g., serotonin, GABA, dopamine), affecting neural signaling and cognitive-behavioral functions. Additionally, alterations in microbial metabolites (such as reduced butyrate among SCFAs and elevated ammonia levels) compromise the BBB and induce neurotoxicity, contributing to ASD-like symptomatology. Consequently, potential preventive strategies include modifying maternal diet during pregnancy and lactation (avoiding high-sugar/salt/fat and ultra-processed foods while increasing dietary fiber intake), supplementing with probiotics/prebiotics to restore microbiota balance and enhance gut barrier function, and optimizing infant formula with HMOs to mimic the microecological regulatory effects of breast milk. Notably, current research emphasizes the need for large-scale clinical trials to validate the efficacy of maternal probiotic supplementation in reducing offspring ASD risk. Future studies should prioritize elucidating the precise interactions between specific microbial metabolites and fetal neurodevelopment, and developing personalized dietary interventions based on maternal gut microbiota profiles. These findings lay a crucial foundation for developing novel ASD prevention strategies targeting the maternal gut-fetal brain axis.
